# Characterization of pulmonary function in Duchenne Muscular Dystrophy

**DOI:** 10.1002/ppul.23172

**Published:** 2015-03-09

**Authors:** O.H. Mayer, R.S. Finkel, C. Rummey, M.J. Benton, A.M. Glanzman, J. Flickinger, B.‐M. Lindström, T. Meier

**Affiliations:** ^1^ Division of PulmonologyThe Children's Hospital of PhiladelphiaPhiladelphiaUSA; ^2^ Division of NeurologyNemours Children's HospitalOrlandoFlorida; ^3^ Santhera PharmaceuticalsLiestalSwitzerland; ^4^ PharmaTurkuFinland; ^5^ Divisions of NeurologyThe Children's Hospital of PhiladelphiaPhiladelphiaUSA; ^6^ Department of Physical TherapyThe Children's HospitalPhiladelphiaUSA

**Keywords:** Muscular dystrophy, peak expiratory flow, forced vital capacity, pulmonary function test, natural history

## Abstract

Decline in pulmonary function in Duchenne Muscular Dystrophy (DMD) contributes to significant morbidity and reduced longevity. Spirometry is a widely used and fairly easily performed technique to assess lung function, and in particular lung volume; however, the acceptability criteria from the American Thoracic Society (ATS) may be overly restrictive and inappropriate for patients with neuromuscular disease. We examined prospective spirometry data (Forced Vital Capacity [FVC] and peak expiratory flow [PEF]) from 60 DMD patients enrolled in a natural history cohort study (median age 10.3 years, range 5–24 years). Expiratory flow‐volume curves were examined by a pulmonologist and the data were evaluated for acceptability using ATS criteria modified based on the capabilities of patients with neuromuscular disease. Data were then analyzed for change with age, ambulation status, and glucocorticoid use. At least one acceptable study was obtained in 44 subjects (73%), and 81 of the 131 studies (62%) were acceptable. The FVC and PEF showed similar relative changes in absolute values with increasing age, i.e., an increase through 10 years, relative stabilization from 10–18 years, and then a decrease at an older age. The percent predicted, FVC and PEF showed a near linear decline of approximately 5% points/year from ages 5 to 24. Surprisingly, no difference was observed in FVC or PEF by ambulation or steroid treatment. Acceptable spirometry can be performed on DMD patients over a broad range of ages. Using modified ATS criteria, curated spirometry data, excluding technically unacceptable data, may provide a more reliable means of determining change in lung function over time. **Pediatr Pulmonol. 2015; 50:487–494.** © 2015 Wiley Periodicals, Inc.

## INTRODUCTION

Duchenne Muscular Dystrophy (DMD, OMIM 310200) is the most common neuromuscular disorder of childhood.^1^ Due to mutations in the DMD gene, a deficiency in dystrophin protein causes premature muscle cell failure and leads to progressive muscle atrophy.^2^, ^3^ This causes weakness, loss of ambulation and motor skills, and ultimately pulmonary and cardiac failure that typically results in death in the third decade of life.^4^ Respiratory insufficiency in DMD is caused by progressive respiratory muscle failure, in particular the diaphragm, which leads to restrictive respiratory disease and added burden on the respiratory system.^5^ While respiratory morbidity is heralded by a decrease in lung volume (vital capacity; VC), in time a patient loses the ability to inhale and exhale fully, to cough effectively, and finally to ventilate properly. This predictably leads to the need for airway clearance and mechanical ventilation in the latter second to third decades.^5^, ^6^

Regular assessment of pulmonary function starting late in the first decade of life is part of the current standard of care in DMD.^6^ This monitoring has typically included annual assessments of lung volume (forced vital capacity (FVC)), and measurements of respiratory muscle strength (maximal inspiratory (MIP) and expiratory (MEP) pressures). When there is concern for early respiratory failure, a full polysomnogram is performed to assess for nocturnal hypoventilation and often to initiate ventilatory support.^7^

A maximal expiratory maneuver, with full inspiration followed by a complete exhalation, is required to properly perform spirometry and produce an accurate FVC. Progressive respiratory muscle failure, as in DMD, limits the ability of patients to inhale and exhale fully and forcefully, causing the FVC to decrease and produce a restrictive respiratory pattern.^8^, ^9^ Although FVC is an important outcome measure to follow the progression of respiratory disease in DMD, it can also be influenced by scoliosis especially in nonambulatory DMD patients.^10^, ^11^

Peak expiratory flow (PEF) is a measure of the maximal or peak flow produced during an exhalation with maximal effort, and as such is the most effort‐dependent measure of lung function. While often used as a measure of airway obstruction in patients with asthma, assessment of PEF may also be helpful as a measure of disease progression in DMD since it assesses maximal expiratory effort as a surrogate measure for expiratory muscle strength.^12^ PEF should mirror the longitudinal change in FVC, since decrease in the expiratory force (PEF) should occur coincidently with a decrease in both the depth of maximal inspiration (MIP) and ability to forcefully exhale (MEP) to produce a lower FVC.

One of the potential problems in using PEF and FVC as a measure for pulmonary status in DMD is that they are volitional and among the most effort dependent measurements of lung function. However, in insuring that this testing is done with maximal effort and is technically sound, both measures have the potential to be very useful in integrated measures of respiratory function. Furthermore, in being done in a clinic setting by trained therapists, as opposed to a separate pulmonary function testing laboratory, FVC and PEF can be very practical in the measures of lung function.

We felt that the current American Thoracic Society (ATS) guidelines for acceptability of spirometry were not appropriate for a patient with DMD, and would be extremely hard to meet. The standard ATS guidelines are made to maximize expiratory flow rate to the point of flow limitation in order to show that maximal flow characteristics of airways. This is extremely important for testing to evaluate for obstructive lung disease. In DMD and in other neuromuscular disease the overriding concern is of vital capacity, and how it changes over time with respiratory muscle weakness and decreased depth of breathing. The force with which vital capacity is exhaled is of little consequence; however, the volume exhaled is extremely important. Acknowledging this, we developed modified criteria for acceptability based on current ATS guidelines for spirometry acceptability and evaluated the percent of subjects able to perform acceptable spirometry over time.

Currently, it is difficult to plan clinical intervention studies using FVC or PEF as outcome measures in DMD, since there is little, if any guidance in the literature on the reliability or precision of spirometry testing in this population.^4^, ^11^, ^13^, ^14^, ^15^, ^16^, ^17^, ^18^, ^19^, ^20^, ^21^ We hypothesized that spirometry could be performed reliably in subjects with DMD, and with the precision required to describe an accurate longitudinal trend in FVC and PEF that could then be used to design outcome studies.

## PATIENTS AND METHODS

Prospective data in DMD patients were collected in the Neuromuscular Clinic at The Children's Hospital of Philadelphia (CHOP, Philadelphia, PA) from 2005–2010, as part of an Institutional Review Board approved United Dystrophinopathy Project (UDP) genotype–phenotype cohort study, in which spirometry was performed and the data were collected. All English‐speaking patients with a DMD phenotype and confirmed dystrophin mutation were invited to participate in this study. Subjects signed an informed assent, based upon age, and their parents signed an informed consent. No specific intervention was provided. The DMD care consideration guidelines, though not published until 2009, were generally followed.^6^, ^22^Daily glucocorticoid therapy (prednisone 0.75 mg/kg/day, maximum 30 mg/day, or deflazacort 0.9 mg/kg/day, maximum 36 mg/day) was recommended to subjects at the discretion of the treating physician, typically at ages 4–7 when the boy was in the “plateau phase”. The high dose weekend regimen was utilized in a small number of patients and the “10 day on/10 day off” regimen was not used.

For purposes of this study, DMD was defined clinically as onset of symptoms by age 5, a positive Gowers' sign, abnormal gait pattern, markedly elevated creatine kinase level and the loss of ambulation by age 12.^23^ Ambulation was defined as the ability to walk unassisted and without braces for at least 10 m.

Pulmonary function testing (PFT) was performed during regular Neuromuscular Clinic visits at CHOP for subjects of age 5 years and above using a Renaissance II spirometer (Puritan Bennett, Boulder, CO, 34% of data) or a KoKo spirometer (nSpire, Longwood, CO, 66% of data) with both instruments delivering comparable PFT results (as determined by visual comparison). PFTs were obtained by experienced physical therapists within the Neuromuscular Clinic (AG, JF) who were trained in performing spirometry as part of the UDP study group to perform testing in compliance with ATS guidelines.^24^ A minimum of three trials with maximal effort were attempted by each subject, and the therapist performing the testing made the initial determination if the effort was acceptable. Expiratory flow volume curves that were clearly technically unacceptable were not saved for analysis. Data were then reviewed for acceptability by one investigator (OHM), a pediatric pulmonologist with clinical experience in caring for patients with DMD and who directs the Pulmonary Function Testing Laboratory at CHOP. The criteria for acceptability that were used was modified from ATS criteria to maximize the quality of the data but acknowledge the limitations of a patient with respiratory muscle weakness (Table 1). Expiratory flow‐volume curves and standard spirometry data, including FVC and PEF, were printed for each subject, with patient identifiers then removed. PFT data were normalized for age, gender, race, and height. The NHANES III normative equations were used to obtain percent predicted values for FVC (FVC%) and PEF (PEF%).^25^ Once the subject could no longer stand, the larger of sitting arm span or recumbent segmental length (head to hip, hip to knee, and knee to foot) was used to calculate a surrogate measure of height. For some subjects this surrogate measurement was less than the prior standing height, in which case the last valid height measurement was used and carried forward in order to not have a spurious increase in FVC% or PEF% simply because of the decrease in the predicted value. The use of corticosteroid medication and other clinical data was entered into the UDP database. A subject was defined as being off systemic glucocorticoid therapy if he was off therapy for at least 30 days.

**Table 1:** Modified American Thoracic Society (ATS) Criteria for Acceptability for Spirometry

• No delay in the onset of exhalation of expiratory flow‐volume curve.
• Clearly defined peak flow.
• Termination of expiratory flow at or below 10% of peak flow.
• Plateau of the volume‐time curve (no change of >25 mL over the final 1 sec of exhalation).
• Reproducibility in FVC with agreement within 10% between the highest two FVC values.
• No minimum forced expiratory time.

Data were analyzed (CR, BML) using TIBCO Spotfire (version 4.0.1.8, TIBCO, Palo Alto, CA) for graphical analysis and descriptive statistics. The annual rates of change for PEF% and FVC% were calculated using a random regression coefficient model, where the slope was estimated by age.

## 3. Results

### Subject Demographics

A total of 60 subjects (51 Caucasian, 5 African‐American, 2 Hispanic, and 2 Asian) covering an age range between 5.0 and 24.1 years (median 10.3 years) were included in the study (Table 2). At the time of their first visit, 27 subjects (45.0%) were being treated with glucocorticoids (age: median 8.9 years, range: 5.1–16.4 years), of which 16 (59.3%) were using prednisone/prednisolone and 11 (40.7%) were taking deflazacort. The subjects not on steroid therapy were significantly older (median 12.2 years; range: 5.0‐24.0 years) compared to steroid treated patients (*P* = 0.003, Student's *t* test). The subjects not on steroids includes subjects who were never on glucocorticoids (based on the decision of the treating neurologist or refusal of the caregiver) and subjects for whom steroid medication had been discontinued. Mean duration of steroid treatment prior to the first PFT assessment was 34.6 months (SD: 24.5), as reported in the medical history of 25 subjects. Ten subjects had previously taken steroids for a mean of 26.5 (SD: 24.4) months but were in the “not on steroid” cohort. Six subjects started and one subject discontinued steroid treatment during the study period. During the course of this study, the treating physician sometimes reduced subjects' steroid dosages in an effort to temper side effects, in which case the minimum prednisone dosage was typically 0.3 mg/kg/day and deflazacort 0.4 mg/kg/day. A few subjects were adjusted to the high dose weekend prednisone schedule, generally 10 mg/kg/week.

**Table 2:** Subject Demographics

Number of patients:	
Total	60
Those with acceptable data	44
Number of visits:	
Total	131
Those with acceptable data	81
Age [y]^*^	11.6 ± 4.9 (10.3, 5.03–24.1)
Weight [kg]^*^	37.9 ± 17.3 (16–95)
Height [cm]^*^, ^#^	135.5 ± 21.6 (99–184)

^*^ Mean ± SD (median, min–max), taken from all patients (N = 60)

^#^ Derived from standing height, sitting arm span or recumbent segmental length (see Methods)

Thirty‐eight subjects (63.3%) were ambulatory at their first visit and 4 subjects (mean age: 12.2 years) became nonambulatory during follow‐up visits (2 subjects were on steroids the entire time, 1 subject was off steroids both before and after losing ambulation and 1 subject started steroid treatment at the time when he lost ambulation).

The distribution of visits, proportion of subjects being treated with steroids, proportion of ambulatory subjects, and valid PFT assessments by age group are shown in Table 3. For subjects under age 8, 75% were using steroids and all were ambulatory. The proportion of subjects who were ambulatory decreased with increasing age, to the point that all subjects were nonambulatory after 16 years of age.

**Table 3:** Visit Statistics

Age	Number of subjects^*^	Number of visits	(%) Visits with subjects on steroids	(%) Subjects ambulatory	(%) Valid PFT observations
<6	4	4	75.00	100.00	50.00
6–8	15	21	76.19	100.00	66.67
8–10	17	22	81.82	95.45	59.09
10–12	20	26	69.23	88.46	65.38
12–14	13	17	52.94	35.29	70.59
14–16	11	12	50.00	25.00	58.33
16–18	9	12	33.33	0	58.33
18–20	6	7	0	0	57.14
20–22	4	5	0	0	40.00
22<	3	5	0	0	60.00
ALL	60	131	56.49	58.54	61.83

^*^ Subjects can contribute data to several age groups

### Analysis of Pulmonary Function Tests (PFTs)

**Figure 1:**
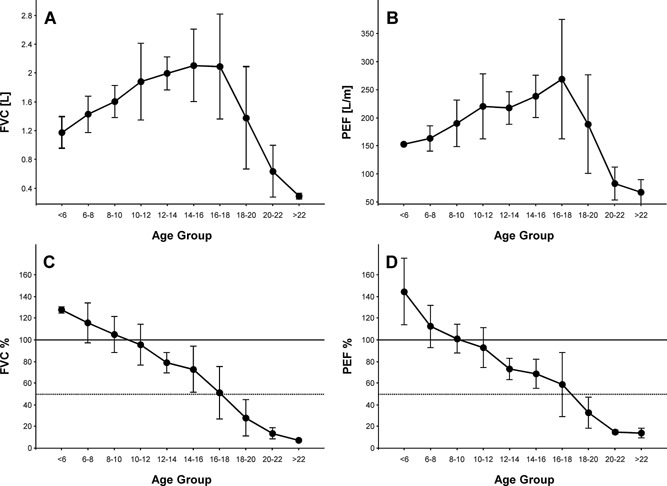
Forced Vital Capacity [FVC], in liters (A); Peak Expiratory Flow [PEF], in liters/minute (B); percent predicted FVC (C); and percent predicted PEF (D) for patients with PFTs fulfilling the modified ATS criteria. Data are mean ± SD. N = 2–17 per data point. For reference, the horizontal lines depict 100% (solid) and 50% (dotted) of predicted FVC and PEF.

In order to maximize data integrity, modified ATS guidelines for acceptability of spirometry (Table 1) were used to determine acceptability. From the 131 PFTs collected across all age groups, 81 PFTs (61.8%) from 44 patients were considered acceptable. The 6–16 year‐old subjects performed acceptable spirometry 63.7% of the time, compared to only 55.2% for the subjects over 16 years of age. Both FVC (L) and PEF (L/min) changed in three phases with increasing age. Up to approximately 10 years of age, subjects showed a nearly linear increase in FVC (Fig. 1A) and PEF (Fig. 1B), followed by a period of relative stabilization through 18 years, after which there was a rapid decline. However, FVC% (Fig. 1C) and PEF% (Fig. 1D) declined almost linearly from the 6–8 years of age cohort through the 20–22 years of age cohort.

**Figure 2:**
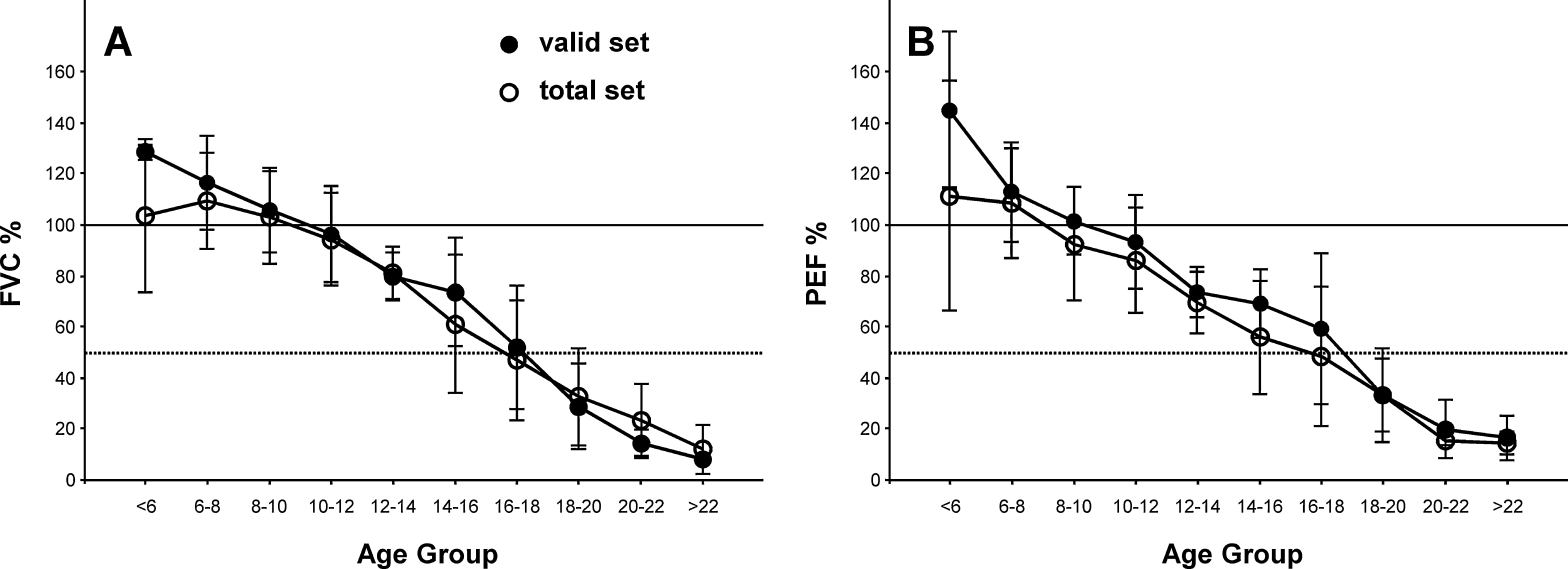
Comparison of all subjects' test results and the curated valid data set for percent predicted FVC and PEF. Data are mean ± SD. N = 2–12 per data point. For reference, the horizontal lines depict 100% (solid) and 50% (dotted) of predicted FVC and PEF.

The annual rate of change for FVC% was −5.0 ± 0.7%/year and for PEF% was −5.8 ± 0.6%/year (mean ± SE) as calculated by a random coefficient regression model, with similar rates of change for the data sets with only acceptable (valid) data and that with all (both acceptable and unacceptable trials) data (Fig. 2). The total data set with both acceptable and unacceptable data had a slightly larger standard deviation when compared to the data set with only acceptable data (PEF%: 21.2% vs. 17.3%; FVC% 19.0% vs. 17.3%), but the mean did not differ significantly at any age group.

**Figure 3:**
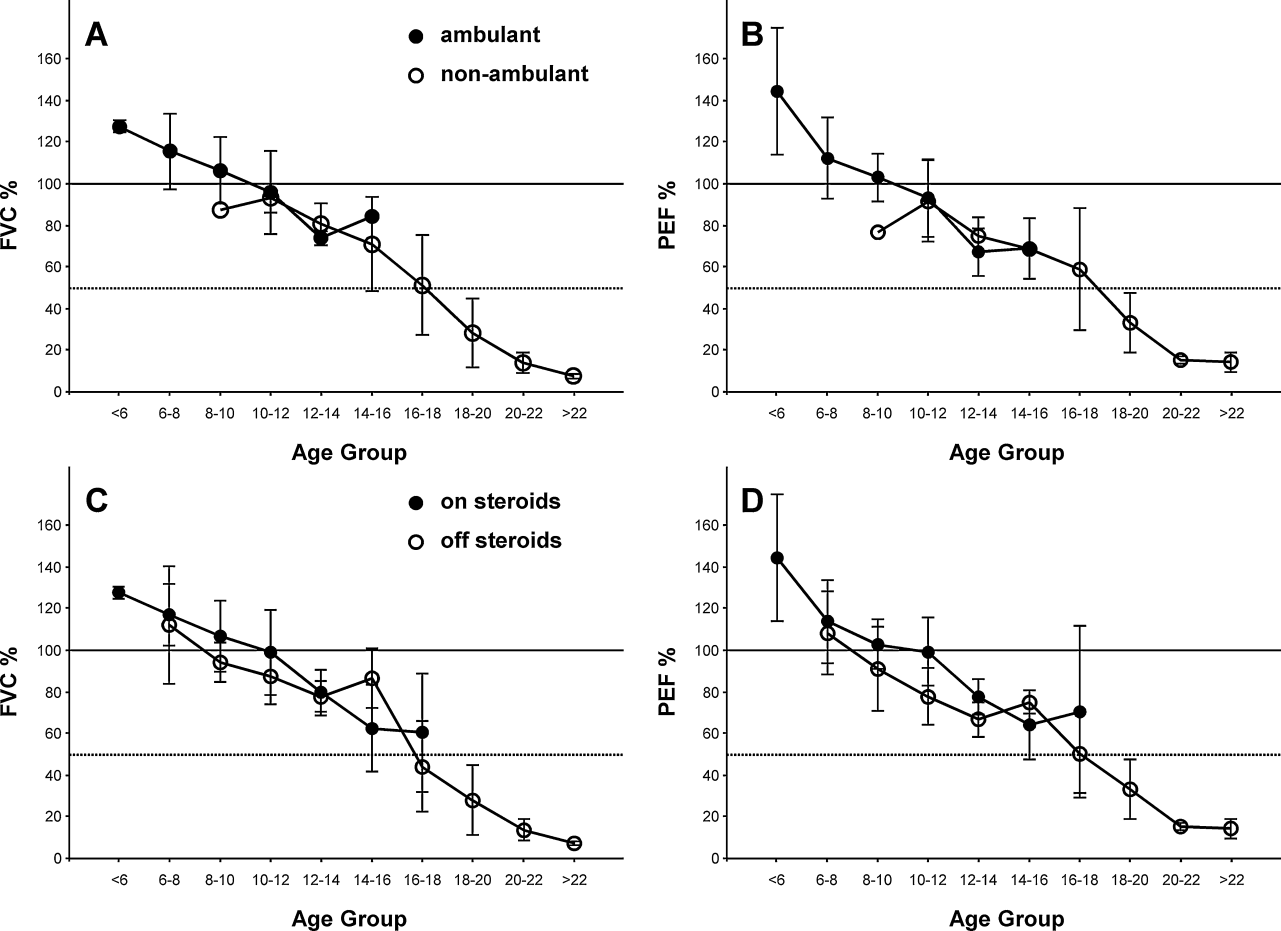
Pulmonary Function Test data separated by ambulatory and steroid status. The percent predicted PFT data are separated by ambulatory status [FVC (A) and PEF (B), N =  1–15 per data point] and by glucocorticoid usage [FVC (C) and PEF (D), N = 2–17 per data point]. Data are mean ± SD for all patients fulfilling the ATS criteria for an acceptable study. For reference, the horizontal lines depict 100% (solid) and 50% (dotted) of predicted FVC and PEF.

In the 8–16 year old cohort, when subjects began to lose ambulation, FVC% and PEF% were comparable between the ambulatory and nonambulatory subgroups (Fig. 3A and B). Specifically, the approximate linear decline in FVC% and PEF% seen in the ambulatory subgroup was unchanged when patients became nonambulatory.

When PFT data were separated by systemic glucocorticoid use, the change in FVC% and PEF% decreased at comparable rates, for the subgroup of subjects using steroids compared to the subgroup not using steroids (Fig. 3C and D).

## DISCUSSION

We hypothesized that acceptable spirometry could be performed through the late teenage years in patients with DMD using modified acceptability criteria that were more appropriate for patients with DMD. We further hypothesized that success in properly performing spirometry would decline with age. Using modified ATS criteria for acceptability, subjects between 5 and 24 years of age were able to produce acceptable spirometry between 50% and 71% of the time. The acceptability decreased with increasing age through teenage years, where respiratory failure typically begins. Considering the physical demands required to perform spirometry properly, the decrease in the rate of acceptable studies with advancing age corresponds well to the loss of ambulation and suggests a link between the ability to perform spirometry and broader functional deterioration.

It is critical to apply strict criteria for acceptability of spirometry to ensure that the data obtained are reliable and, therefore, an accurate measurement of lung function. Without this precision the spirometry data may not accurately reflect the decline in lung function and the increased variability may limit the comparability of data sets obtained from different studies. Furthermore, it is also important to apply criteria that acknowledge the limitations of a subject with DMD, but still are rigid enough to insure that the data reported are precise and reliable. We feel that the modified criteria are appropriate on both levels, but need to be explored more broadly in a larger study.

Interestingly there were similar rates of change for the data sets with only acceptable data, using modified ATS criteria, and that with combined acceptable and unacceptable data. While this is an unexpected finding, it applies only to the population data as a whole and not to each individual subject. Unfortunately, we did not have enough data to explore this further. Therefore, while applying strict criteria for acceptability may only have a modest impact in minimizing the variability of the overall data set, we speculate that there would be a much larger impact on the precision of intrasubject changes longitudinally in an intervention trial.

The data analyzed show a clear, almost linear decline in FVC% and PEF% across the age range from 5–24 years. This mirrors findings from other published cohorts of subjects with DMD.^17^, ^26^ However, a surprising finding in this study was the observation that the FVC% and PEF% were not significantly different in ambulatory compared to nonambulatory subjects or in glucocorticoid using compared to nonsteroid using subjects. As motor function was significantly better in the steroid treated group (data not shown), the apparent lack of PFT response to steroid therapy may be attributable to the definition used here. The nonsteroid group included some subjects for whom steroids had been prescribed but refused by the caregiver. This subgroup may have been more severely affected than the group for whom steroid therapy had never been recommended and may have artificially lowered the lung function in the nonsteroid group. Also, those subjects who had taken steroids chronically, and possibly benefited, but were discontinued for at least 30 days, may have biased the nonsteroid group data in a positive direction. Unfortunately, due to the number of subjects, we were not able to separate the data well enough to interrogate this issue further.

It is also fair to question whether FVC% or PEF% are sensitive enough measurements to assess progression of muscle weakness. While, decreases in FVC% and PEF% certainly mirror general disease progression as an indirect measure of loss of muscle function, they may not be sensitive enough to demonstrate what might be small changes between ambulatory and nonambulatory patients. Maximal inspiratory and expiratory pressures, static lung volumes, such as total lung capacity (TLC), functional residual capacity (FRC), residual volume (RV), and peak cough flow were not examined in this analysis and may provide additional information and conclusions.

One important consideration when using FVC and PEF data as outcome measures in a clinical trial is the risk of including technically inaccurate data from subjects unable to both inhale and exhale fully with maximal effort. This is more likely to occur in the weaker subjects, and will result in an erroneously low FVC, which may lead to data from weaker subjects being artificially low if these technically inadequate studies are still included. Thus, it is difficult to interpret previously published reports in DMD that use FVC as an outcome measurement of spirometry, especially in those reporting subjects with more advanced weakness (nonambulatory and off steroid cohorts). Without having access to the raw data or a clear idea of the criteria for accepting spirometry trials, it is impossible to know if this was a factor in the studies by Biggar et al.^26^, ^27^

There are several limitations in this study which impact how broadly the conclusions can be applied. Although comparable to other published studies, the number of enrolled subjects (60) was relatively small and was not evenly distributed by age. The length of follow‐up varied and the number of older subjects able to perform spirometry decreased with age making accurate longitudinal analyses challenging. Intrasubject variability in these PFT measures over time was not explored here and needs further analysis in a larger data set.

The data were collected in a neuromuscular clinic setting by physical therapists who were trained on how to perform spirometry properly, and were not as experienced in performing spirometry as full‐time PFT therapists in a PFT laboratory. While it is unclear how much of an impact this had on the data and the acceptability, this study was not designed to make this comparison. We feel that it will be important to examine this prospectively in a future study.

It is also important to recognize that change from standing height to a surrogate measure (arm span or recumbent length) may have intrinsic limitations. Ulnar length and a conversion equation to standing height may remedy this issue.^28^

In summary, it is feasible but challenging to obtain accurate measurements of pulmonary function in the clinic in boys with DMD. The age range captured in this study will likely encompass the ambulatory and nonambulatory patients with DMD enrolled in clinical trials. The data from this study may help in the design of clinical trials in DMD, particularly the estimation of effect size and power calculations, using FVC% or PEF% as outcome measurements.
